# Lumbar Endoscopic Microdiscectomy: Where Are We Now? An Updated Literature Review Focused on Clinical Outcome, Complications, and Rate of Recurrence

**DOI:** 10.1155/2015/417801

**Published:** 2015-11-24

**Authors:** Giulio Anichini, Alessandro Landi, Federico Caporlingua, André Beer-Furlan, Christian Brogna, Roberto Delfini, Emiliano Passacantilli

**Affiliations:** ^1^Department of Neuroscience, Neurosurgery, Imperial College of London, Charing Cross Hospital, London, UK; ^2^Department of Neurology and Psychiatry, Neurosurgery, University of Rome “Sapienza”, Rome, Italy; ^3^Department of Neurological Surgery, Wexner Medical Center, The Ohio State University College of Medicine, Columbus, OH, USA; ^4^Department of Neurosurgery, King's College of London, London, UK

## Abstract

Endoscopic disc surgery (EDS) for lumbar spine disc herniation is a well-known but developing field, which is increasingly spreading in the last few years. Rate of recurrence/residual, complications, and outcomes, in comparison with standard microdiscectomy (MD), is still debated and need further data. We performed an extensive review based on the last 6 years of surgical series, systematic reviews, and meta-analyses reported in international, English-written literature. Articles regarding patients treated through endoscopic transforaminal or interlaminar approaches for microdiscectomy (MD) were included in the present review. Papers focused on endoscopic surgery for other spinal diseases were not included. From July 2009 to July 2015, we identified 51 surgical series, 5 systematic reviews, and one meta-analysis reported. In lumbar EDS, rate of complications, length of hospital staying, return to daily activities, and overall patients' satisfaction seem comparable to standard MD. Rate of recurrence/residual seems higher in EDS, although data are nonhomogeneous among different series. Surgical indication and experience of the performing surgeon are crucial factors affecting the outcome. There is growing but still weak evidence that lumbar EDS is a valid and safe alternative to standard open microdiscectomy. Statistically reliable data obtained from randomized controlled trials (better if multicentric) are desirable to further confirm these results.

## 1. Introduction

Endoscopic disc surgery (EDS) is a relatively well-known technique, which has been introduced since the ‘80s, but shows rapidly expanding interest in the last few years. The concept behind it is to provide a minimally invasive approach to the lumbar spine when treating disc herniations. Ideally, the goal of the developing endoscopic disc surgery is to get the same results obtained using standard microdiscectomy, providing effective treatment, targeted to the nerve decompression and not only focused on pain relief, like in nerve root/peridural injections, but at the same time avoiding discomfort related with open techniques.

Although fascinating, results of this technique are still debated, mostly due to (1) learning curve for surgeons not confident with the endoscopic kit in a spinal environment; (2) rate of recurrences of symptoms/radiological finding, which still seems to be higher compared to standard microdiscectomy; (3) lack of consistent evidence comparing outcomes of endoscopic and microscopic discectomy.

We performed an extensive review of the literature about EDS. The review is focused on introduction and development of the technique over time, results in terms of outcome, recurrence, and complications rate, available evidence reporting comparison between EDS and standard microdiscectomy, and possible future development.

## 2. Historical Background

First series of EDS are reported from the late ‘80s. Kambin and Schaffer reported initially successful experience in 88% of patients undergoing percutaneous discectomy [1] and similar results after the introduction of the endoscope in the so-called arthroscopic discectomy [2]. Between the end of the ‘80s and the beginning of the ‘90s other authors reported similar results [3–7], with a variable success rate being variable (65–85% of “good results”). All these series reported a combination of posterior-lateral or far lateral approach to the disc through the lateral foramen. This is performed under radiological guidance, with subsequent introduction of cannulated system and endoscope for disc fragment removal.

Consequent diffusion of the technique led to extended series, reported in the mid ‘90s. With growing surgical experience, several authors started to raise and assess criticisms related with the far lateral percutaneous approach. The main problem concerned the lack of improvement in radicular symptoms, requiring reexploration surgery in 7 to 11% of cases [8–11]. Moreover, as pointed out by Kim and Park in a comparative review, the percutaneous discectomy through a far lateral approach might be limited by anatomical factors, such as presence of iliac crest, large facet joint, or L5 transverse process [12]. To overcome these problems, endoscopic interlaminar approach was subsequently developed and popularized by several authors [13–16]. This is performed by a posterior approach to the disc space from the standard microdiscectomy route, through a window obtained by positioning the cannula into the interlaminar space and removing the disc fragment after opening of the ligamentum flavum.

## 3. Surgical Technique

As mentioned above, EDS has been broadly practiced, and many variations in name and techniques have been reported. Terminology is quite variable and, as always, different names indicating the same procedure with few variations are reported. However, to sum things up we might say that EDS mainly include two different approaches. The first one is the one we define here as the* transforaminal approach*; possible variations of this name include far lateral endoscopic approach, posterior-lateral endoscopic approach, and arthroscopic far lateral/posterior-lateral approach. The second one is the* interlaminar approach* described by Ruetten et al. [14]. Indications and technique for these approaches are different, and they both require thorough preoperative evaluation.

It is not our intentions to describe the surgical technique in detail, since authoritative textbooks and papers already report it [17]. However, the main basic steps and few important nuances are reported in the following paragraphs.

### 3.1. Transforaminal Approach

**Figure 1:**
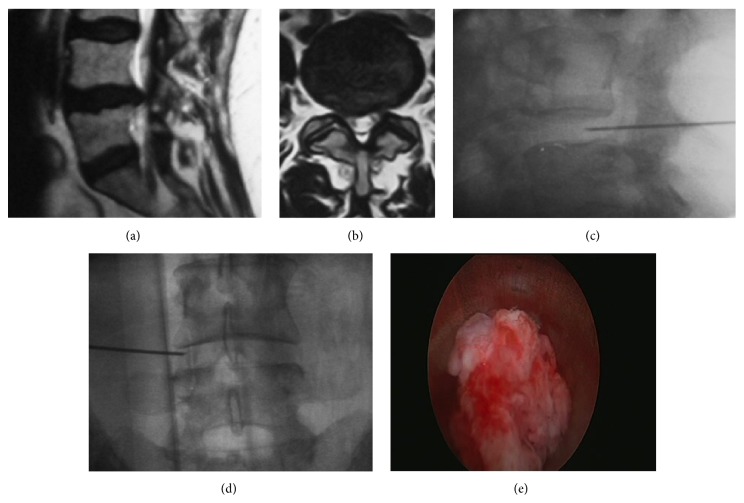
(a-b) Sagittal and axial T2-weighted MRI images of an L4-L5 right disc bulging causing foraminal stenosis. Approach is performed through an entry point located ~7 cm from the midline. (c-d) Intraoperative fluoroscopy and lateral and anteroposterior (AP) projections showing the position of the instrumentation. (e) Fragment of the disc removed from the endoscopic cannula.

*Indications*. Indications are intraforaminal disc herniations, extreme lateral/far lateral/extracanalar disc herniations, lateral disc herniations in selected cases, and confidence in the technique (Figure 1).

*Contraindications*. Contraindications are L5-S1 segment (iliac crest and/or L5 transverse process are obstacles for surgical route), anatomical variations, large median and paramedian disc herniations/cauda equina syndrome (decompression not achievable through this route), caudally or cranially migrated fragments, and elderly patients with stenosis-like picture (even if only on the recess).

*Advantages*. Minimally invasive approach, lower degree of muscle manipulation/damage, reduced postop back pain, reduced postop fibrosis (both muscle and periradicular), and limited bone decompression prevent risk of postop instability due to excessive removal of facet joint, direct visualization of decompressed root from its extracanalar route.

*Disadvantages*. Disadvantages are need for experience, learning curve for surgeons used to standard microdiscectomy, progressively more limited movements as the foramen is entered, and no possible treatment for L5-S1 level and median disc herniations.

*Surgical Technique*. Standard operative conditions are obtained. While usually performed under general anaesthesia for better comfort of both the surgeon and the patient, use of local anaesthesia might be helpful to localize the nerve root intraoperatively. C-arm covered with sterile drape is mandatory throughout the whole procedure. Surgeons, nurses, radiology, and anaesthetics team must wear appropriate protection. Patient is positioned on a standard frame, taking into account not to cause abdominal compression, which might increase venous bleeding. Skin entry point is localized empirically between 10 and 12 cm from the midline; further lateralization might be required in heavyweight patients (Figure 3). Continuous fluoroscopic guidance is used to introduce an 18-gauge needle and to check its position in both anterior-posterior (AP) and lateral projections (Figure 1). The aim of the needle is the triangular working zone, an area of the extracanalar space defined superiorly by the existing root and ganglion, inferiorly by the disc itself, and medially by the lateral margin of the facet joint. In AP projections, pedicle is ideally divided into three lines: lateral, mid, and medial pedicular lines [17]. Ideal positioning of the tip of the needle should be at the level of the mid pedicular line in anterior-posterior projections and inferior margin of the foramen in lateral projection, parallel to the superior end plate of the inferior vertebral body. At this point, needle is replaced with a wire; then skin incision is made around it and the wire is used as a guide to introduce cannula. Cannula is then maintained against the disc fibres and continuous washing of saline through the cannula is used to continuously clean the surgical view. Ideally, a washing system should be integrated with the cannula. Different angle endoscopic optics (30°, 45°, 70°, and 90°) can be used for the inspection and discectomy. Once the endoscope is inserted, discectomy or fragment removal is performed using dedicated forceps, also provided with different angles, which allow resection in all possible directions. Lateralization of the entry point allows achieving more medial exposure and resection and even removing bulging located into the spinal canal. From this point of view, several variations of the approach have been described, including bilateral and unilateral biportal approaches [18–20], all used to obtain different degree of exposure and discectomy. Once the decompression of the nerve root is satisfactory, haemostasis is performed and instruments are removed. Skin is closed with one or two stitches.

### 3.2. Interlaminar Approach

*Indications*. Indications are prolapsed median or paramedian disc herniations, recess/lateral canal stenosis, and synovial cysts.

*Contraindications*. Contraindications are intra- or extraforaminal disc herniation, lumbar stenosis, and spinal instability at the same segment.

**Figure 2:**
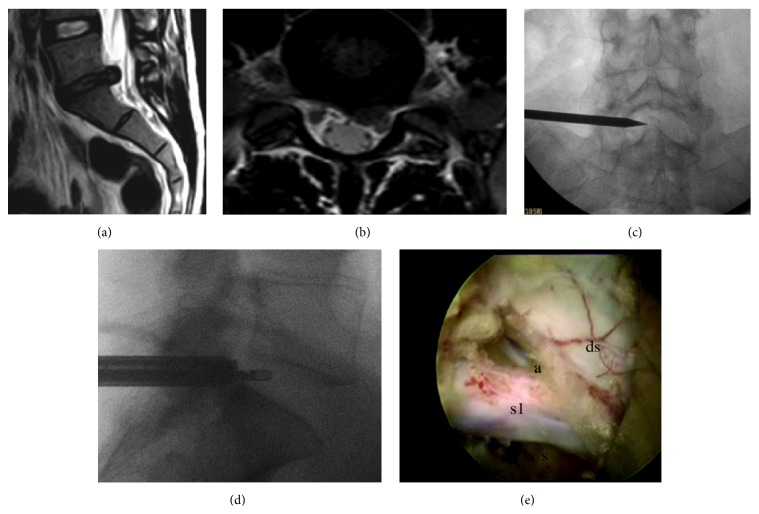
(a-b) Preoperative T2-weighted sagittal and axial MRI showing an L5-S1 disc herniation impinging the left S1 nerve root. (c-d) Intraoperative fluoroscopy showing different phases of the transforaminal approach. AP view: pointer showing the L5-S1 interlaminar window and lateral view showing the radiofrequency bipolar endoscopic probe inside the L5-S1 intervertebral disc. (e) Removal of the herniated disc material has been completed. At the end of the procedure, the dural sac (ds), the S1 nerve root (s1) with its axilla (a), and shoulder (s) can be clearly visualized. Taken from the senior author's personal series.

*Advantages*. Best access to L5-S1 disc space (as compared with the transforaminal approach), lower degree of muscle manipulation/damage, reduced postop back pain, reduced postop fibrosis (both muscle and periradicular), and limited bone decompression prevent risk of postop instability due to excessive removal of facet joint (Figure 2).

*Disadvantages*. Disadvantages are being still not ideal for spinal stenosis, need for experience, learning curve for surgeons used to standard microdiscectomy, and higher rate of recurrence.

**Figure 3:**
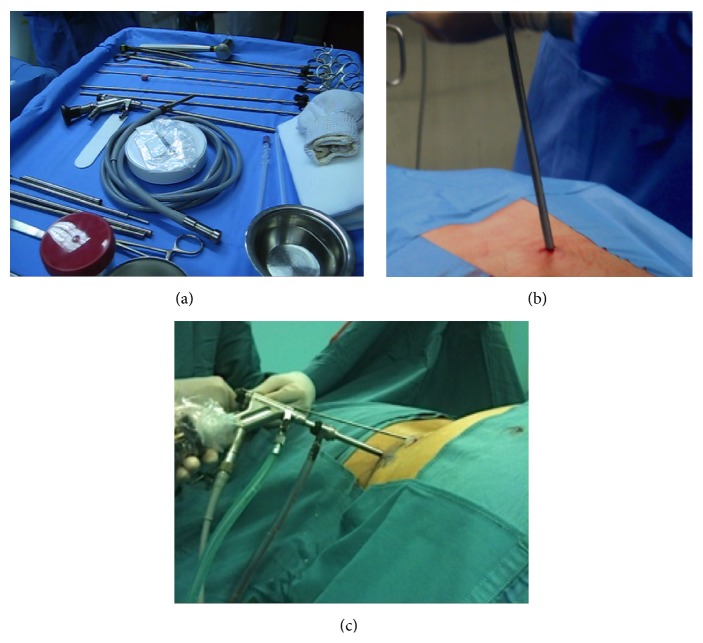
(a) Instrumentation: light source at the center of the surgical table, endoscopic cannula, and different pituitary forceps adapted for endoscopic use on the top of the picture. (b) Positioning of the endoscope during an interlaminar approach. (c) Positioning of the endoscope during a transforaminal approach, extreme lateral variation.

*Surgical Technique*. Endoscopic access is determined under fluoroscopic AP guidance; skin incision is made as medial as possible in the craniocaudal midline of the interlaminar window (Figure 3(b)). A dilator is inserted bluntly toward the lateral edge of the interlaminar window as far as the flavum ligament. Dilator must have an oblique direction from the midline, to the lateral edge of the flavum ligament to permit endoscopic access under the zygapophyseal joint. The subsequent part of the operation is performed under lateral fluoroscopic guidance. An operating sheath is inserted with beveled opening directed toward the flavum ligament. Direction in lateral fluoroscopic view must be pointed towards the disc space with the instruments end just upon the facet joint. Dilator is removed and the endoscope is inserted. The further procedure is performed under visual control and constant irrigation. All the endoscopic instruments and radiofrequency bipolar system pass through the working channel. The flavum ligament is clearly exposed with the aid of radiofrequency bipolar and forceps. A lateral incision is made, approximately 5 mm long, up to the zygapophyseal joint. With lateral fluoroscopic guidance being possible to have an easy craniocaudal orientation, medial to lateral orientation is obtained reaching the facet joint and touching bone with instruments and dissector (Figure 2). Bone of the ascending facet and superior lamina can be partially resected, thus obtaining a wide exposure of the descending facet. Opening is enlarged using burrs and endoscopic bone punch. After entering the spinal canal the flouting epidural fat is clearly visible; neural structures are exposed. After having clearly recognized the passing nerve root and the dural sac, the operating sheath with beveled opening serves as a second instrument to protect and gently manipulate the neural structures in order to expose and remove the disc herniation. In order to avoid neural damage, particularly in the cranial segment, prolonged lateral displacement of the passing root must be avoided. Traction is performed on intermittent basis only after having clearly gained medial to lateral orientation inside the spinal canal. If gently lateral traction cannot be achieved, drilling of the descending facet can be considered in order to gain more space and achieve a first indirect decompression. At the end of the procedure the passing nerve root must appear clearly decompressed with the fatty lubricating tissue floating around the nervous structures (Figure 2(e)). It is possible to gently retract medially the passing nerve root with a blunt dissector; just make sure all prolapsed disc fragments have been removed.

## 4. Materials and Methods

There is extensive literature about EDS and multiple surgical series are reported. Many reviews have also been published, although not systematic in most cases, and few clinical trials comparing EDS with standard microdiscectomy.

It was not the purpose of this paper to perform an extensive and omnicomprehensive literature review. For these reasons, with few exceptions, literature review is focused on the last six years. We reviewed all English-written papers about lumbar spine endoscopic microdiscectomy. Papers were collected using PubMed Database, and keywords for Medline were “endoscopic lumbar discectomy”. Literature reviews, case series, meta-analysis, randomized controlled trials, case-cohort studies, and prospective and retrospective series were all included. Small series (<10 cases) and case reports were excluded. Series focused on new techniques, disc recurrences, spinal instability, or different techniques were not considered, although some of them are mentioned in the Discussion.

## 5. Limitations

Literature review was limited to English-written papers and only included the last 6 years of publications; thus it is not intended as systematic review or meta-analysis or as a comprehensive review about this topic.

## 6. Results

### 6.1. Case Series

From July 2009 to July 2015, we found 51 series about lumbar endoscopic discectomy reported in the international literature. Main results of each series are reported in Table 1 [21–28, 30–61, 63, 65–72].

Out of this group, 21 articles reported results of surgical series, 5 papers were focused on analysis of surgical technique and its variations, 4 were comparison between endoscopic discectomy and standard microdiscectomy, 5 were focused on complications, and the rest were focused on different topics (learning curve, use of annuloplasty, etc.), Table 1 [21–28, 30–61, 63, 65–72].

Number of patients enrolled varied from 15 [26] to 400 [40]. Most common scales used for assessment and outcome were Visual Analog Scale (VAS), Oswestry Disability Index (ODI), and MacNab criteria. Several Asian authors also used Japanese Orthopedic Association (JOA) scale.

Surgical technique was not always specified, but larger series of patients treated through interlaminar approach were growing through the years. Specifically, both Yadav and Kaushal reported 400 and 300 patients treated through interlaminar approach, respectively [40, 51]. However, this should not be misleading. Indications for transforaminal and interlaminar approaches became more defined over the years. Transforaminal approaches were used mostly for far lateral, foraminal, and extraforaminal disc herniations. Several variations of this technique were reported, including the possibility of reaching the spinal canal by enlarging the discectomy from outside the spinal canal, thus improving the working channel [18, 19]. This approach was partially abandoned with the advent of interlaminar approach, which made it possible to remove even medially located disc fragments. Today, choosing the different approach mostly depends on the experience of the surgeon and accurate selection of patients. As exposed in Table 1, recent series are reporting patients treated with both techniques, but different indications.

Outcomes reported are quite homogeneous among most series. Virtually all authors report a good to excellent outcome in 70 to 90% of patients treated, according to MacNab criteria. Rate of recurrence/residual is by far one of the most debated topics in literature. Interestingly, most series reported a rate of recurrence similar to standard microdiscectomy (2 to 10%). However, results are extremely variable from this point of view. One of the largest series [40] reported 2 patients over 400 showing recurrence at follow-up (0,2%), the other one reporting 10% rate of recurrence on 344 patients [42]. Kulkarni and Sencer reported 1,5% and 5%, on 188 and 163 patients, respectively. Most patients of these series were treated through interlaminar approach, thus ideally comparable with standard microdiscectomy. One large series of patients treated through transforaminal approach reported rate of recurrence of 20% [52], and similar amount was reported by Wang et al. in a series comparing two different surgeons at a different stage of their learning curve [61]. Rate of complications (CSF leak, dysesthesia, nerve root damage, etc.) is quite homogeneous in all series.

The overall opinion reported in discussion/conclusions sections of most authors is that results of endoscopic microdiscectomy are comparable to the one of standard microdiscectomy. Out of this group, two series report considerations worth mentioning. The first is the one from Teli and colleagues, who reported a higher rate of complications in patients treated with endoscopic discectomy (224 patients, randomized in 3 groups) [65], the second being the one from Lee et al., who reported significant reduction of low back pain in patients treated through endoscopic technique (54 patients, nonrandomized).

### 6.2. Literature Reviews: Systematic Reviews and Meta-Analyses

In early 2015, Dohrmann and Mansour published one of the largest reviews analysing results of different surgical techniques for lumbar disc herniations. Outcomes of multiple studies were reviewed and compared. Good to excellent outcome is reported in 80% of patients undergoing endoscopic discectomy. These results were similar to the standard microdiscectomy (70 to 84%) [73]. Despite being based on the largest cohort of patients collected from international literature (39.000 overall), this review was based on extremely nonhomogeneous studies, and therefore it did not discuss further important data regarding endoscopic microdiscectomy, such as the rate of complications, recurrence, issues related to indications, and learning curve.

The main problem with the data analysis is the lack of systematic reviews, this being also related to lack of randomized control trials comparing standard microdiscectomy/open discectomy with endoscopic lumbar discectomy.

In the last 6 years of medical literature, we found 6 reviews overall, including the one from Dohrmann et al., 2 of them being Cochrane reviews [73–78].

Smith and colleagues reported a detailed selection of studies over a 6-year period, in order to identify randomized control trials comparing endoscopic discectomy with microdiscectomy [Smith]. Out of 109 studies analysed, the authors found only 4 randomized controlled trials meeting the eligibility criteria [58, 65, 79, 80]. As expected, no significant outcome differences were noted between standard microdiscectomy and endoscopic discectomy. However, Teli and colleagues series reported higher rate of complications in patients undergoing endoscopic discectomy. This study has obviously a deep impact on this analysis, being one of the largest randomized series reported.

Another interesting review is the one reported by Birkenmaier and colleagues [78]. This found 5 randomized control trials [80–84], all of them reporting similar results about endoscopic discectomy: (1) reduced hospital staying and quicker return to work following endoscopic procedures; (2) lower rate of complications in endoscopic series; (3) similar rate of recurrences observed in either of the two techniques. However, this review included also cervical endoscopic discectomy series, and it did not include the previously mentioned series from Teli et al. [65], which reported different results.

Two Cochrane reviews were also reported [74, 75]. The first one, by Gibson et al., systematically reviewed quality and results of randomized and quasi-randomized trials of the surgical management of disc prolapse [75]. This included a variety of different techniques, including standard microdiscectomy, endoscopic discectomy, and chemonucleolysis. Results did not show strong clinical evidence supporting percutaneous techniques. The second one, by Rasouli et al., specifically compared randomized and quasirandomized control trials of standard microdiscectomy techniques and all minimally invasive techniques, including endoscopic microdiscectomy and tubular microdiscectomy [74]. Analysis was focused on outcome in terms of pain relief and functional results, as well as on all related data, such as length of hospital admission, rate of complications, and rate of recurrence. The authors reported weak evidence that minimally invasive techniques were associated with a slightly higher risk of recurrence and worse outcome, but with lower risks of complications related with the procedure [74].

All the previously mentioned reviews reported that more randomized control trials are needed in order to get stronger evidence about endoscopic lumbar discectomy.

Finally, one meta-analysis was reported so far in the international literature [85]. This included 9 randomized controlled trials (most of them already mentioned above) and compared their results. In terms of length of hospital staying, overall patient satisfaction, outcome as measured with MacNab criteria, and minor blood loss, the overall rate of good outcome seems to be higher in EDS, although with different degrees of statistical significance. Even here, however, the authors stressed the need for more randomized controlled trial and the fact that the evidence supporting these results is still not strong, despite this being probably the more statistically reliable study published so far.

## 7. Discussion

This review has serious limitations and, as specified before, it should not be intended as a systematic or comprehensive review of all studies reported in the literature. Our goal was only to provide an update about this topic, focusing on the main debated issues (recurrence/complications rate) and on possible future developments.

What we know today is that the number of centres and surgeons practicing EDS is exponentially increasing. Despite its basics being described since the early ‘90s, in the last ten years we have assisted at a wide diffusion and rapidly growing spreading of this technique. As mentioned previously, 51 surgical series have been reported in the English literature, and far more were found in other languages. Moreover, we focused our attention only on transforaminal and interlaminar endoscopic discectomy, also excluding recurrence series and series focused on a specific aspect.

One of the largest series of EDS reported has been published in 2015 and includes 10228 patients treated through a transforaminal approach [86]. The authors reported an incomplete removal in 2,8% of cases and recurrence rate of 0,8%; both these two types of data, taken alone, are comparable to those reported on standard microdiscectomy series. Remarkably, the authors focused their attention on the rate of incomplete removal and recurrences related to the learning curve of the surgeons and the inappropriate positioning of the surgical instruments, which have been found to be the main factors influencing negative outcome in this particular study. These have been also stressed by several series reported in Table 1 and they seem to be one of the crucial points of the debate around EDS. In fact, we might also speculate that all different results might be related to different indications and different experience of the reporting surgeon(s).

Proper choice of indication is of paramount importance for the outcome. In authors' experience and on the basis of the literature data, endoscopic techniques should be used in patients showing fresh or relatively fresh fragments, even migrated, with minor or no signs of diffuse spinal degenerative disease, such as broad disc bulge, spinal stenosis secondary to hypertrophic ligament/osteophytes, and spinal instability. Moreover, use of the endoscope in spinal procedures may be challenging for surgeons not used to the endoscopic kit and techniques, and it requires dedicated training and learning curve. Two series recently reported highlighted the different results obtained from surgeons with different level of experience in EDS. Specifically, both articles reported higher rate of recurrence/residual in patients operated on by surgeons at the earlier stage of their learning curve [44, 61]. However, the majority of largest series reported in the last 6 years showed results comparable to those of standard microdiscectomy, with growing number of authors describing even better results in terms of postop pain and return to work (Table 1).

However, the lack of randomized controlled trial keeps us cautious about the interpretation of these results. Ideally, a multicentred, randomized control trial enrolling large number of patients and surgeons with similar degree of experience should clarify whether results of EDS are comparable or superior to the ones of standard microdiscectomy.

## 8. Conclusions: What Is Next?

Despite the lack of defined clinical evidence, lumbar EDS is undoubtedly a rapidly expanding field and it is not unreasonable to look at its future developments as incredibly promising. Even if not mentioned here, indications for endoscopic techniques are gradually extending to other lumbar diseases, such as instability [29], multilevels surgery [87], recurrent discs [88], and spinal stenosis [89, 90].

Basing on the data available so far about lumbar EDS, few points are highlighted.

1. There is growing but still not sufficient evidence that lumbar EDS shows slightly better results in terms of minor tissue damage, shorter hospital staying, quicker return to normal daily activities, and patient satisfaction.
2. Rate of recurrence/residual is still a matter of debate, and it seems to be strictly related to appropriate surgical indications and level of training of the operating surgeon.
3. Rate of complications seems similar in both open and endoscopic techniques; however results reported are extremely nonhomogeneous in different series.
4. More randomized controlled trials, systematic reviews and meta-analysis are needed to clarify whether lumbar EDS can be considered comparable if not superior to standard open discectomy or not.

## Figures and Tables

**Table 1:** Series reported in the literature in the last 6 years. FED: full endoscopic discectomy; MED: microendoscopic discectomy; EDS: endoscopic discectomy; MD: microdiscectomy; TF: transforaminal; IL: interlaminar; OC: outcome; DVT: deep venous thrombosis. ^*∗*^When reported alone, values are referred only to as recurrence rate.

First author	Year	Study	Number of pts.	Techn.	OC measures	Outcome	Recurrence rate/residual/redo^*∗*^	Complications
Li [21]	2015	EDS, comparison between FED and MED	65	TF and IL	VAS and ODI	No differences, shorter hospital staying in FED group	8,6% FED; 6,7% MED	1 dural tear
Türk [22]	2015	Surgical series EDS	105	TF	VAS and ODI	90,4% pain relief	2 redo surgeries	Not mentioned
Wang [23]	2015	Surgical series EDS	207	TF	VAS, ODI, and MacNab	71–86% excellent OC, age related	3 to 5% age-related	3 dural tears, 1 postop instability
Li [24]	2015	Surgical series EDS	72	IL	VAS, ODI, and MacNab	97% good to excellent OC	1	No complications noted
Sairyo [25]	2014	Surgical series EDS, analysis of complications	100	TF and IL	—	—	—	2% nerve injury; 1% postop hematoma
Liao [26]	2014	Surgical series EDS	15	TF	VAS and MacNab	93% good to excellent OC	—	—
Sencer [27]	2014	Surgical series EDS	163	TF and IL	VAS and ODI	88% good to excellent OC	8 (5%)	6 (3%) dural tears; 5 (2,9%) types of postop worsening
Yoshimoto [28]	2014	Surgical series EDS, comparison between far lateral and intraforaminal disc herniations removal	25 (far lateral) + 93 (IL)	TF	VAS and JOA	No significant differences in pain relief between the two groups	—	—
Jasper [29]	2014	Surgical series EDS, comparison between transforaminal and interlaminar approaches	41	TF and IL	VAS and MacNab	75% pain relief in both groups	—	No complications noted
Xu [30]	2014	Surgical series EDS, analysis of learning curve	36	IL	VAS	Excellent outcome	2 pts. converted to open surgery	No complications noted
Hussein [31]	2014	Comparison between EDS and MD	185	IL	NRS, MacNab, and ODI	Statistically significant pain relief in both groups	2; 8 converted to open surgery	3 dural tears
Kulkarni [32]	2014	Surgical series EDS	188	IL	VAS and ODI	Statistically significant pain relief	3 (1,5%)	11 (5%) dural tears, 1 (0,5%) infection, and 1 (0,5%) wrong level
Choi [33]	2013	Surgical series EDS, comparison between transforaminal and interlaminar approaches	30	TF and IL	VAS and ODI	Shorter recovery time in interlaminar	3,3% TF; 6,7% IL	6,7% postop dysesthesia
Wang [34]	2013	Surgical series EDS, comparison between early and delayed surgery	145	—	VAS and MacNab	No significant differences in pain relief between the two groups	8 to 11% redo	No complications noted
Kim [12]	2013	Surgical series EDS, comparison between interlaminar approach alone and interlaminar + annular sealing	224	IL	VAS and ODI	Statistically significant pain relief in both groups	5% IL + sealing; 13% IL alone	—
Yoshimoto [35]	2013	Surgical series EDS	25	—	JOA	80,4% of pain improvement	0	No complications noted
Jasper [36–38]	2013	Surgical series EDS	195	TF	VAS	83,9% improvement in single level pathology; 69,7% improvement in multilevel	—	—
Wang [39]	2013	Surgical series EDS, analysis of learning curve (comparison between 2 groups operated on by surgeons with different level of training)	120	TF	VAS and JOA	Significant improvements in both groups	20 residuals, 14 (23%) group A; 6 (10%) group B; 2 recurrences	2 postop infections
Choi [33]	2013	Surgical series EDS, intraop magnetic imaging	89	TF	VAS, ODI, and MacNab	Significant improvement	4 (4,5%) residuals; 2 (2%) recurrences	2 postop hematomas
Jasper [36–38]	2013	Surgical series EDS	50	TF	VAS	71 to 75% pain relief	10%	No complications
Yadav [40]	2013	Surgical series EDS	400	IL	VAS and MacNab	90% significant improvement	2 (0,5%)	3 facet injuries; 7 dural tears; 2 infections; 1 persistent paresthesias
Soliman [41]	2013	Surgical series EDS	41	IL	VAS and ODI	95% excellent to good improvement	1	2 dural tears
Matsumoto [42]	2013	Surgical series EDS, analysis of recurrences	344	—	JOA	75 to 83% recovery rate	37 (10,8%)	—
Hsu [43]	2013	Comparison between EDS and MD	59	TF and IL	VAS and ODI	No significant differences between EDS and standard microdiscectomy groups	2 recurrences, 4 persistent symptoms	2 nerve root injuries
Chaichankul [44]	2012	Surgical series EDS, analysis of learning curve	50	TF	VAS	Significant improvement in both groups, higher in later stages of learning curve	—	—
Kim [45]	2012	Surgical series EDS for migrated discs	18	IL	MacNab	89% of complete removal	2 residuals	1 dural tear
Hirano [46]	2012	Surgical series EDS	37	TF and IL	VAS and JOA	Significant improvement	2	—
Yoon [47]	2012	Surgical series, comparison of EDS and tubular-retractor microdiscectomy	37 EDS + 35 MD	TF	VAS, ODI, and SF-36	No significant differences between EDS and standard microdiscectomy groups	1 in each group	1 dural tear; 1 bowel perforation
Wang [48]	2012	Surgical series EDS	151	—	MacNab	91% good to excellent OC	5 (3,5%)	5 pts. (3,5%) dural tears; 3 pts. (2,1%) discitis
Lübbers [49]	2012	Surgical series EDS	22	TF and IL	VAS, ODI, and MacNab	18 pts. (81%) good OC	2 (9,1%)	1 stroke
Han [50]	2012	Surgical series EDS, analysis of technique	41	TF	MacNab	39 pts. excellent to good OC	—	2 nerve root injuries
Kaushal [51]	2012	Surgical series EDS	300	IL	MacNab	90% excellent to good OC	—	6 discitis cases; 5 dural tears; 2 nerve root injuries
Kim [45]	2012	Surgical series EDS, analysis of technique	30	IL	—	Significant improvement	—	No complications noted
Tenenbaum [52]	2011	Surgical series EDS, analysis of technique, complications, and learning curve	124	TF	VAS and ODI	OC comparable to open surgery	20,9% redo surgery	1,6% complication rate
Chumnanvej [53]	2011	Surgical series EDS	60	IL	MacNab	91,6% excellent outcome	2	No complications
Cho [54]	2011	Surgical series EDS, analysis of complications	154	TF	VAS and ODI	Significant improvement	3 (1,95%)	1 dural tear; 1 discitis
Choi [55]	2011	Surgical series EDS, focused on annuloplasty and LBP improvement	52	TF	VAS and ODI	78,4% improvement	18 residuals; 2 recurrences	No complications noted
Chen [56]	2011	Surgical series EDS, focused on anesthesia	123	IL	VAS and ODI	Significant improvements in both groups	3	1 dural tear
Dezawa [57]	2011	Surgical series EDS, focused on technique	30	IL	—	Significant improvement	1 persistent radiculopathy	—
Garg [58]	2011	Comparison between EDS and MD	112	TF	ODI	Statistically significant pain relief in both groups	1	EDS, 5 dural tears
Doi [59]	2011	Surgical series EDS	17	TF and IL	JOA	16 pts. significant improvement	3	No complications noted
Casal-Moro [60]	2011	Surgical series EDS	120	TF and IL	VAS and ODI	92% good to excellent OC	7,5% redo surgery	4,1% dural tear; 4 nerve root injuries; 1 DVT; 1 discitis
Wang [61]	2011	Surgical series EDS, analysis of learning curve	30	IL	VAS	Significant improvement	20% converted to open	12 to 10%, depending on the group
Lee et al. [62]	2010	Surgical series EDS	25	TF	VAS and ODI	Significant improvement	1 residual; 1 recurrence	No complications noted
Ahn [63]	2010	Surgical series EDS, focused on annuloplasty and LBP improvement	87	TF	VAS, ODI, and MacNab	72% good to excellent OC	13 converted to open	No complications noted
Jhala and Mistry [64]	2010	Surgical series EDS	100	IL	MacNab	91% good to excellent OC	4	4 discitis cases; 1 nerve root damage
Teli [65]	2010	Comparison between EDS and MD, focused on complications	224	—	VAS, ODI, and SF-36	Higher rate of complications in EDS group	8	6 dural tears; 2 nerve injuries; 1 discitis
Peng [66]	2010	Surgical series EDS	55	—	VAS, NASS, and SF-36	Significant improvement	5%	—
Lee [67]	2009	Comparison between EDS and MD	54—25 EDS, 29 MD	TF	VAS and ODI	Significant improvement in both groups, but reduction in hospital staying and recurrence rate in EDS group	1 EDS persistent pain; 4%	1 unspecified complication
Chae [68]	2009	Surgical series EDS, analysis of technique	153	TF	VAS and MacNab	94% excellent to good OC	Not reported	1 paravertebral hematoma; 3 transient pareses; 8 transient hypoesthesia cases
Zhou [69]	2009	Surgical series EDS	275	TF	MacNab	91% good to excellent OC	5	5 dural tears; 3 infections
